# *Update *on epidemiology of HCV in Italy: focus on the Calabria Region

**DOI:** 10.1186/1471-2334-14-S5-S2

**Published:** 2014-09-05

**Authors:** Nadia Marascio, Maria Carla Liberto, Giorgio Settimo Barreca, Emilia Zicca, Angela Quirino, Angelo Giuseppe Lamberti, Giovanna Bianco, Giovanni Matera, Lorenzo Surace, Giuseppina Berardelli, Lidia Surace, Vincenzo De Maria, Francesca Giancotti, Rosa Anna Leone, Vilma Villella, Salvatore Nisticò, Annelisa Borelli, Vincenzina Caruso, Massimo Calderazzo, Gianfranco Griffo, Rosanna Masciari, Pasquale Minchella, Lucio Cosco, Carmelo Laganà, Angela Oliva, Giuseppe Foti, Maria Teresa Fiorillo, Giuseppa Lo Bocchiaro, Pasquale Surace, Anna Rita Ciccaglione, Massimo Ciccozzi, Francesco Cesario, Carlo Torti, Alfredo Focà

**Affiliations:** 1 Institute of Microbiology, Department of Health Sciences, "Magna Graecia" University, Catanzaro, Italy; 2 Clinic of Infectious Diseases and Hepatology, General Hospital, Lamezia Terme, Italy; 3 Unit of Infectious Diseases, General Hospital, Lamezia Terme, Italy; 4 Unit of Hepatology, University Teaching Hospital, Pisa, Italy; 5 Unit of Hepatology, Hospital "Mater Domini", Catanzaro, Italy; 6 Microbiology and Virology Unit, General Hospital, Lamezia Terme, Italy; 7 Microbiology and Virology Unit, "Pugliese-Ciaccio" Hospital, Catanzaro, Italy; 8 Unit of Infectious Diseases, "Pugliese-Ciaccio" Hospital, Catanzaro, Italy; 9 Microbiology and Virology Unit, "Bianchi Melacrino Morelli" Hospital, Reggio Calabria, Italy; 10 Unit of Infectious Diseases, "Bianchi-Melacrino-Morelli" Hospital, Reggio Calabria, Italy; 11 Microbiology and Virology Unit, Polo Sanitario Nord ASP 5, Reggio Calabria, Italy; 12 National Institutes of Health, ISS, Rome, Italy; 13 Unit of Infectious Diseases, "Annunziata" Hospital, Cosenza, Italy; 14 Unit of Infectious Diseases, Department of Medical and Surgical Sciences, "Magna Graecia" University, Catanzaro, Italy; 15 University Unit of Infectious Diseases, University of Brescia, School of Medicine, Brescia, Italy

## Abstract

The epidemiological profile of HCV infection is evolving in Europe, as well as in Italy. We have previously showed genotype distributions and their dynamics in 2,153 HCV RNA positive patients living in Calabria, Southern Italy, over 11 years. In this study, we extend and update this information by evaluating a hospital-based cohort of 945 HCV RNA positive patients attending five hospitals in the Calabria Region from January 2011 to August 2013. We assessed rates of HCV genotypes according to age and gender and the dynamics of HCV genotype distribution over the 3-year period studied. Data showed that genotype 1b is the most prevalent, followed by subtypes 2a/2c and genotype 3. Genotype 4 exhibited an increase between 2011 and 2013. Also, we found a significant decrease in the median age of subjects infected with HCV genotype 3 and 4 during the period studied. Since HCV genotypes are important in epidemiology, pathogenesis and response to antiviral therapy, a continuous epidemiological surveillance is needed.

## Introduction

Hepatitis C is a viral infection (HCV) of the liver and is one of the major causes of chronic liver diseases including complications such as cirrhosis and liver cancer [1]. The increases of cirrhosis and hepatocellular carcinoma (HCC) over time in HCV infected individuals are of particular concern [2]. So, the burden of HCV for patient health, society and health-care systems is substantial.

About 150 million people worldwide suffer from chronic HCV infection [3]. In Europe, an estimated 8 million people (1.3%) are infected by HCV [4], but a heterogeneous pattern of prevalence has been reported. Indeed, while intermediate-to-high prevalence rates were reported in Eastern and Southern Europe, Western and Northern Europe reported low prevalence rates (≤1.0%). This emphasizes the importance of HCV infection, particularly in these parts of the world [5]. Also, the distribution of HCV genotypes shows a great heterogeneity, reflecting the differences in epidemiology, including modes of transmission and ethnic variability in different countries. Globally, genotype 1, especially type 1b virus, causes approximately 90.0% of infections, while types 2, 3 and 4 are less represented [6]. HCV genotypes 1 and 2 are universally distributed, whereas HCV genotypes 4, 5, 6 and 7 are confined to more specific geographical areas [7].

It has to be recognized, however, that surveillance systems for HCV are prone to underestimation due to the generally asymptomatic nature of acute infections, the marginalization of at risk populations or simply a lack of informative campaigns and testing for HCV. So, not only is data on the incidence of HCV infection limited, but data on its prevalence and subtype distribution is also incomplete [8].

In this paper, we report the contents of the presentation given at the third Workshop of the Regional Study Group on HCV in the Calabria Region (Southern Italy). In this presentation we reviewed prevalence and genotype distribution of HCV in Italy. Also, data from the Calabria Region were reviewed with a focus on special populations (migrants and intravenous drug users, IVDUs). Original and unpublished data updating previous estimates are presented from a hospital-based cohort of 945 HCV RNA positive patients attending five Hospitals in the Calabria region (January 2011 to August 2013).

## Current knowledge and limitations on general HCV epidemiology in Italy

In November 2012, during World Hepatitis Day, the Italian Ministry of Health confirmed that prevalence of HCV seropositivity was higher in Southern and Insular areas (about 8.0%) than in Central and Northern regions (about 2.0%). However, the reports on which this statement were based are outdated or were obtained in limited areas [9-13]. Also, an active surveillance program for symptomatic acute hepatitis cases reported a rate of 0.2/100,000 inhabitants in 2010. Although this is likely to be underestimated because it is well known that HCV infection is rarely symptomatic during its acute phase [14].

Risk factors for HCV infection in Italy have been traditionally related to nosocomial and healthcare related sources. Intravenous drug use, beauty treatment, hospitalization, surgical intervention, dental therapy and having more than two sexual partners were in decreasing order the most frequently reported risk factors [15]. However, the epidemiology of HCV infection has undergone substantial changes over the past two decades, with a progressive decrease in incidence and a shift in risk factors [16]. In particular, the most recent decrease in incidence rates could be due to changes in injecting behavior among IVDUs and information campaigns on HIV/AIDS. It has yet to be seen whether a reduction of information campaigns on HIV/AIDS will contribute to further resurgence of HCV in IVDU and/or in people with risky sexual behaviors.

Migration into Italy is a recent and growing phenomenon. It will influence prevalence of infections but data are still very limited. Indeed, in Italy, relatively high prevalences of HCV infection was detected by Eurostat 2008 in migrants coming from the following States: Albania 9.5%, Romania 4.9%, Morocco 1.1% [17]. A study conducted by Surveillance Systems of ECDC (European Centre for Disease Prevention and Control) reported 26,678 cases of HCV infection between 2007 and 2010; this corresponds to a rate of 6.93 per 100,000 population [18].

The Travel and Migration Medical Centre in Lamezia Terme (Catanzaro area, Italy) set up a screening program in migrants and refugees from North Africa (so called North Africa Emergency Program) in the Calabria Region. Herein we present major evidence from this program. Between July 2011 and June 2012, 1,278 immigrants arrived in the Calabria Region. Of the 1,050 adults and 228 children included, the top three countries of origin were: Somalia (20.0%), Nigeria (18.0 %), and Ghana (10.0%). Among these individuals, 767 consented to be screened and 32 (4.2%) had chronic HCV infection (2 immigrants were HCV-HIV co-infected and 3 were HCV-HBV co-infected). Age distribution of the HCV infected people was: 6.2% under 15 years, 75.0% between 16 and 30 years, 15.6% between 31 and 45 years, and 3.2% above 45 years. More data and continuing surveillance are needed in the incoming migrants to understand whether and how persistence of HCV infected migrants and secondary transmission of HCV in the host population could modify current trends of the epidemics, prevalence of HCV subtypes and their prevalence distribution.

## Prevalence and distribution of HCV genotypes

The overall prevalence of HCV RNA positive subjects in Italy is about 3.0% and more than 50.0% of these are older than 65 years [19]. These patients were the platform for HCV genotyping studies, but even these data are spotted and restricted to limited populations.

Amongst Italian IVDUs, Sereno et al. [20] reported that the 3a HCV subtype was the most prevalent (41.3%), followed by subtypes 1a (23.1%) and 1b (20.6%). However, significant changes in the relative prevalence of genotypes have occurred since 1965: genotype 3 infections decreased from 48/116 (41.4%) in 1965-1985 to 22/84 (26.2%) in 1986-2006. Prevalence of genotype 4 was significantly higher in patients infected after 1985 compared to patients infected before this year.

Petruzziello et al. [21] assessed variations in the distribution of hepatitis C virus (HCV) genotypes in the metropolitan area of Naples, Italy. The authors observed that subtype 1b (48.3%) and genotype 2 (31.7%) were prevalent in older patients, whereas genotypes 3a and 1a were observed more frequently in the younger population. Genotype 1b was particularly common in females. Moreover, by comparing data observed from 2009 to 2011 with data related to a sample of 176 HCV RNA positive patients collected from 2006 to 2008, a cohort effect emerged, with increasing prevalence of genotype 1b among the elderly.

In the Calabria Region, we conducted an 11-year surveillance on HCV genotypes. During the 2001-2011 period, subtype 1b was found to be the most prevalent (49.2%) followed by subtype 2a/2c (22.4%). Genotype 3 was the third most frequent (7.4%), whereas genotype 4 showed a rate of 6.2%. Interestingly, the prevalence of genotype 4 has increased in recent years, while median age of patients with HCV-4 appeared to decrease in 2006-2011 compared to 1997-2001 and 2001- 2005 periods [22,23].

## Update on HCV genotypes in the Calabria region

To better define epidemiology of HCV genotypes in the Calabria Region, here we report an analysis in the most recent years, following the same methodology of the previous studies, and including further geographical areas that were not previously considered. The update of our epidemiological analysis includes 945 HCV RNA positive patients attending five hospitals from January 2011 to August 2013. Samples from 547 patients living in the Catanzaro area and attending University *"Magna Græcia" *Hospital, *"Pugliese-Ciaccio" *Hospital, and "Giovanni Paolo II" Hospital were collected. We also report a sample of 398 patients living in Reggio Calabria area and attending *"Bianchi Melacrino Morelli" *Hospital and "Polo Sanitario Nord Azienda Sanitaria Provinciale 5". The study was approved by *"Magna Graecia" *University Ethical Committee of Catanzaro, in compliance with Declaration of Helsinki. For this retrospective, non-pharmacological study, informed consent has not been provided by patients since it is not deemed to be necessary by Italian legislation.

HCV RNA from serum samples was extracted by Cobas AmpliPrep and detected by Cobas TaqMan HCV test (Roche Diagnostics, Milan, Italy). Versant HCV Genotype 2.0 Assay (Siemens, Healthcare Diagnostic Inc., Tarrytown, NY, USA) was performed according to the manufacturer's instructions. All patients were born in Italy, 550 (58.2%) of them were male and 395 (41.8%) were female.

Distribution of HCV genotypes in the 945 HCV RNA positive patients attending clinical centers in Calabria Region stratified by gender is shown in Table 1. Subtype 1b was the most prevalent (37.5%) followed by subtype 2a/2c (22.4%). As previously observed [23] HCV 1b was not gender related. By contrast, from January 2011 to August 2013, subtype 2a/2c was found mainly in female patients (45.7% male versus 54.3% female, P < 0.05 by χ^2 ^test). Genotype 3 was the third most frequent (11.8%) and was significantly more common in male patients (83.9% male versus 16.1% female, P < 0.05 by χ^2 ^test). Genotype 4 showed a rate of 7.5%, with male versus female difference (67.2% versus 32.8%, P = 0.0712 by χ^2 ^test). Such significance index would suggest a statistical trend in this test. Therefore, here found a further increase of genotype 4 (Table 1) with respect to the previous studied period 2001 - 2011 (6.2 %) [23].

**Table 1:** HCV genotypes/subtypes distribution and gender of 945 patients from January 2011 to August 2013 in Calabria region.


**HCV genotypes/subtypes**	**No. of isolates**	**Percentage (%)**	**Gender**				**P value**

			**Male**	**(%)**	**Female **	**(%)**	

**1**	76	8.0	43	56.5	33	43.5	NS

**1b**	354	37.5	191	53.9	163	46.1	NS

**1a**	73	7.7	47	64.4	26	35.6	NS

**1a/1b**	5	0.5	4	80.0	1	20.0	NS

**2**	42	4.5	26	61.9	16	38.1	NS

**2a/2c**	212	22.4	97	45.7	115	54.3	0.0005

**3**	112	11.8	94	83.9	18	16.1	0.0052

**4**	70	7.5	47	67.2	23	32.8	NS

**5**	1	0.1	1	100.0	-	-	-

**Total**	**945**	**-**	**550**	**58.2**	**395**	**41.8**	**-**

**NS = not significant (P > 0.05)**.

**Figure 1:**
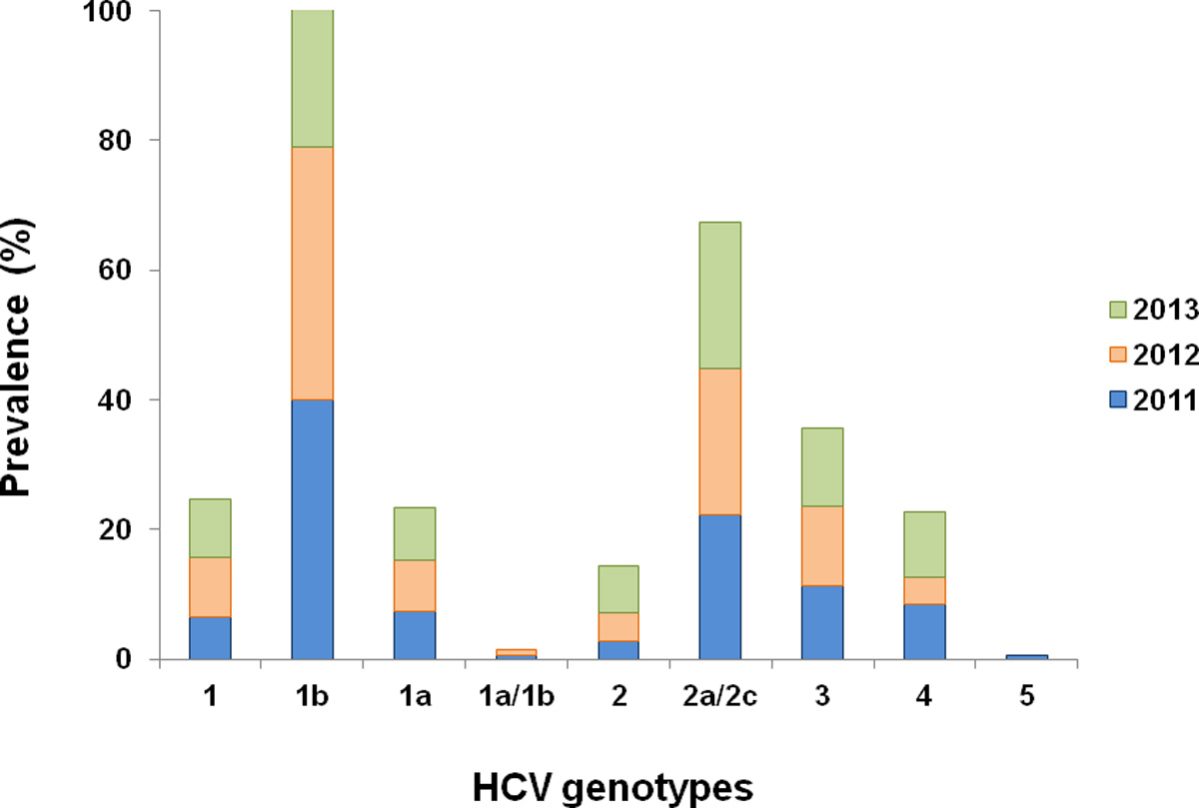
**Dynamic distribution of HCV genotypes/subtypes from January 2011 to August 2013 in Calabria Region**.

**Figure 2:**
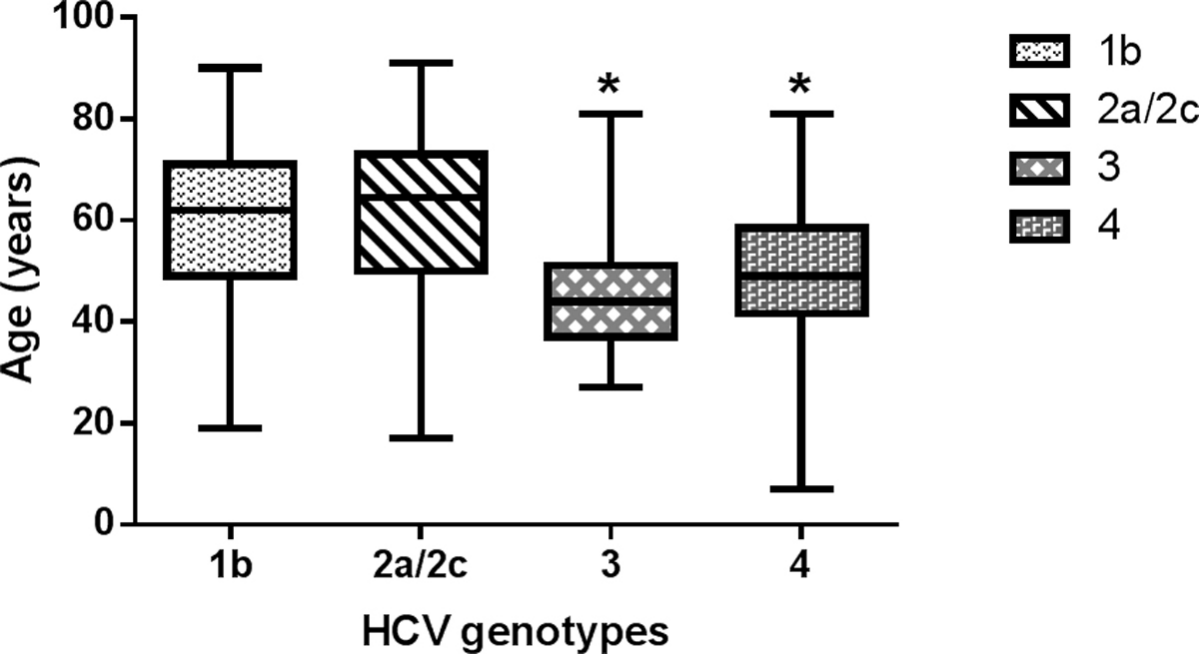
**Box plot of genotypes 1b, 2a/2c, 3 and 4 distribution by HCV patients age in the January 2011-August 2013 period**. * P < 0.01 versus genotypes 1b and 2a/2c (ANOVA plus Tukey test).

The overall dynamics of HCV genotypes in the 945 HCV RNA positive patients in the five healthcare facilities is reported in Figure 1. Subtype 1b decreased from 2011 (40.1%) to 2012 (38.9%) and to 2013 (31.2%), while subtype 2a/2c and genotype 3 did not vary significantly over time. Genotype 4 increased showing a peak of 10.1% during 2013. Statistical analysis of HCV patient age, stratified by genotypes, showed a significant decrease in median age of genotype 3 and genotype 4 HCV infected subjects (P < 0.01 ANOVA plus Tukey test) (Figure 2). Data reported in this study confirm and statistically support previous observations [23,24].

Unfortunately, we could not extrapolate any association between specific HCV genotypes and risk factors for HCV acquisition due to the lack of data. With this objective in mind, we intend to implement our collaboration through the SINERGIE network [25]. In the meantime, spotted observations have been collected. For example, Infectious Diseases Unit and Microbiology and Virology Unit of General Hospital (Lamezia Terme, Italy) seek to determine prevalence and distribution of HCV genotypes in Calabrian IVDUs. Fifty-six patients with chronic hepatitis C were examined between 2011 and 2013. This cohort was composed by 44 males (78.6%) and 12 females (21.4%) of whom 15 individuals (26.7%) were co-infected with HIV. HCV 3a and 1a appeared to be the most frequent genotypes (1:3 each), confirming observations in different settings [20,21].

## Conclusion

From our review, we conclude that epidemiological studies were performed on a limited number of patients, who are not representative of the global Italian population. So, national surveillance programs are necessary. Moreover, further data on HCV prevalence and genotype distribution in migrant populations are urgently needed. In the Calabria Region, subtype 1b, 2a/2c and genotype 3 showed different associations with age and gender. HCV types 1b and 2a/2c were equally distributed among patients over and below 65 years. By contrast, genotype 4 and genotype 3 were more frequent in patients of younger age. Our updated estimations confirm a raise in genotype 4, in particular in the Catanzaro area. Lastly, surveillance performed in the North Africa Emergency Program revealed a chronic HCV infection in about 4.0% of individuals. This surveillance should continue both in autochthonous and migrant populations.

## List of abbreviations used

HCV: Hepatitis C Virus; HCC : Hepatocellular Carcinoma; AIDS : Acquired Immune Deficiency Syndrome; IVDUs : Intravenous Drug Users.

## Competing interests

The authors declare that they have no competing interests.

## Authors' contributions

NM collected data, contributed to data analysis and to manuscript writing; MCL performed design research and wrote the manuscript; GSB, EZ carried out HCV genotyping and collected data; AQ, AGL, GB, GM contributed to data analysis; LS, GB, LS, VDM, FG collected clinical data; RAL, VV, SN carried out HCV genotyping; AB, VC, MC, GG collected clinical data; RM, PM carried out HCV genotyping; LC, CL collected clinical data; AO carried out HCV genotyping, GF collected clinical data; MTF, GLB, PS carried out HCV genotyping; ARC, MC contributed to manuscript writing; FC collected clinical data; CT contributed to data collection and manuscript revision; AF contributed to design research and wrote the manuscript.

## Declarations

The publication of this supplement was partly supported by an unrestricted grant provided by Roche. The articles were independently prepared by the authors with no input from Roche. Roche were not involved in selecting the articles for the supplement.

This article has been published as part of *BMC Infectious Diseases *Volume 14 Supplement 5, 2014: Proceedings of the Third Workshop of the HCV Study Group in the Calabria Region: Results from the South Italian Network for Rational Guidelines and International Epidemiology (SINERGIE) Project. The full contents of the supplement are available online at http://www.biomedcentral.com/bmcinfectdis/supplements/14/S5.
